# Assessing the Potential for Rooftop Rainwater Harvesting from Large Public Institutions

**DOI:** 10.3390/ijerph15020336

**Published:** 2018-02-14

**Authors:** Dagnachew Adugna, Marina Bergen Jensen, Brook Lemma, Geremew Sahilu Gebrie

**Affiliations:** 1 Ethiopian institute of Architecture, Building Construction and City Development, Addis Ababa University, P.O. Box 580, Addis Ababa, Ethiopia; 2 Department of Geosciences and Natural Resource Management, Copenhagen University, København, Rolighedsvej 23, 1958 Frederiksberg C, Denmark; mbj@ign.ku.dk; 3 College of Science, Addis Ababa University, P.O. Box 1176, Addis Ababa, Ethiopia; brklmm2008@gmail.com; 4 Ethiopian institute of Water Resources, Addis Ababa University, P.O. Box 1176, Addis Ababa, Ethiopia; gsahilu@gmail.com

**Keywords:** water deficit, rooftop rainwater harvesting, large public institutions

## Abstract

As in many other cities, urbanization coupled with population growth worsens the water supply problem of Addis Ababa, Ethiopia, with a water supply deficit of 41% in 2016. To investigate the potential contribution of rooftop rainwater harvesting (RWH) from large public institutions, 320 such institutions were selected and grouped into 11 categories, from which 25–30% representative 588 rooftops were digitalized and the potential RWH volume computed based on a ten-year rainfall dataset. When comparing the resulting RWH potential with the water consumption, up to 2.3% of the annual, potable water supply can be provided. If reused only within one’s own institution, the self-sufficiency varies from 0.9 to 649%. Non-uniform rainfall patterns add uncertainty to these numbers, since the size of the storage tank becomes critical for coverage in the dry season from October to May. Despite the low replacement potential at the city level, RWH from large institutions will enable a significant volume of potable water to be transferred to localities critically suffering from water shortage. Further, large institutions may demonstrate how RWH can be practiced, thus acting as a frontrunner for the dissemination of RWH to other types of rooftops. To narrow the water supply gap, considering rooftop RWH as an alternative water supply source is recommended. However, the present study assumed that financial constraints to install large sized storage tanks are considered as a possible challenge. Thus, future research is needed to investigate the cost-benefit balance along with the invention of a cheap storage tank as they may affect the potential contribution of RWH from rooftops.

## 1. Introduction

Water is a basic human requirement which may be sourced from surface water, groundwater, or rainwater [1]. However, available water supply sources are diminishing owing to the population rise, climate change, and pollution, causing a globally acknowledged situation of water scarcity, especially in developing countries [2,3]. The prediction by Seckler et al. [4] that by 2025 one-third of the population in developing countries will face critical water shortages is to some degree refuted by the United Nation (UN). According to its Millennium Development Goals Report 2015, the proportion of people without access to safe water has declined from 24% in 1990 to 9% in 2015, but it should be noted that the wide disparity among countries remains unchanged [5]. The same source also pointed out that nearly half of all people using unimproved water sources live in sub-Saharan Africa, calling for alternative water supply sources [6].

Ethiopia, a sub-Saharan developing country, is the second most populous country in Africa, with a projected population rise from 40 million in 1984 [7] to 94.351 million in 2016 [8]. It has experienced serious annual droughts at a frequency of once every ten years over the last four decades, with a tendency of the frequency increasing to once in five years [9]. A critical water situation exists in Ethiopia’s urban areas as the centralized water supply, which is the only preferred approach, fails to address the needs of all inhabitants due to a mismatch between the high population growth and the extent of existing water supply infrastructure. The water crisis in Ethiopia seems to be worsened due to the effect of climate change and rainfall pattern variation [10]. For example, in 2015, the Ethiopian rainy season stopped in an untimely manner, resulting in a 1 m reservoir level drop (equivalent to 5,850,000 m^3^) in the Lege Dadi water supply dam in Addis Ababa [11], and the lowest mean annual rainfall of 871 mm, some 179 mm below average [12].

Addis Ababa, the capital of Ethiopia and seat of many international organizations including the African Union, is home to 25% of the urban population in Ethiopia [13]. The population is assumed to have increased from 1.423 million in 1984 to 3.434 million in 2016 [8], accompanied by an increase in single residential units, communal high rise apartment blocks (condominiums), and many new industries, all leading to a significant increase in the water demand, and a current water deficit of around 41% [14]. Rainwater Harvesting (RWH) from rooftops may be one of the alternative water supply sources to supplement the potable water demand of Addis Ababa.

A number of studies show RWH to be commonly used as an alternative water supply source both in developed and developing countries such as the United States of America [15], United Kingdom [16], Australia [17], Germany [18], Ethiopia [19], South Africa [20], Sudan [21], Nigeria [22], Brazil [23], Saudi Arabia [24], and Iran [25]. Despite RWH being widely practiced, some concerns and barriers exist. One of the concerns of RWH is the challenge associated with climate change which could reduce the volume of rainwater. A study conducted by [6] in Australia shows that water saving through RWH can be gained in the range of 2–14% of total water consumption and that the reliability of RWH to meet the water demand of a given locality is reduced from 3 to 16% due to the effect of climate change. Still, RWH represents a climate change adaptation measure [26]. Another barrier is revealed in a study conducted in South Africa by [20], reporting key challenges in South Africa associated with RWH to include the lack of a clear legal framework for the adoption of RWH, making it illegal by strict application of the law, the lack of finances to publicize RWH and build necessary infrastructure, and the nonexistence of a nationwide RWH coordinating body to facilitate RWH through approving required tenure beyond private plots. Quality of rooftop RWH represents another challenge [27,28,29]. A study conducted by Wang et al. [30] suggests that the quality of water supply sources can be predicted by relying on modeling techniques. The biggest challenge to widespread RWH, especially in the developing world, may fall at the center of finance, followed by the volume of water depending on erratic rainfall, calling for large water storages [31], the inability to link with the other urban water components, poor public perception and quality, and a lack of commitment from the politicians. 

The present study surveyed the existing large public institutions along with their actual water consumption, and the actual annual water supply of Addis Ababa. Then, the actual water supply of Addis Ababa and the large public institutions were compared with the potential rooftop RWH from the large public institutions to understand its contribution to the city’s and the individual large public institutions’ potable water supply through using the obtainable rainwater. Conversely, previous studies in Ethiopia highlight the various RWH categories and their contribution for various uses (e.g., irrigation, water supply) at country level, e.g., [19].

Large public institutions may play a special part in RWH. They occupy a significant rooftop area, have a rather simple ownership structure with typically a single owner, financial access may be advantageous, and being public they are often exposed to many citizens, and may signal a high level of trust from being part of a larger administration. Thus, large public institutions may provide a big water yield, and serve as frontrunners or demonstrations to scale-up the practices to city level and spread the word for a change in practice that may also reach residential and private institutions’ rooftops. A study in Japan [32] presents a notable example of a public institution serving as frontrunner in RWH. 

Using Addis Ababa as a case representative of rapidly developing cities in the Global South struggling with water supply, the idea of this study was to estimate the potential of RWH to link with the potable water supply, focusing on large institutions. RWH in Addis Ababa is encouraged by a guideline established through a rain water harvesting association in 1999, but practiced only at an insignificant level with few, small-scale traditional practices at household levels for some non-potable applications. This limited practice is the case despite all water demanding activities (e.g., toilet flushing, cloth washing, irrigation, car washing, and cleansing) being dependent on the costly potable water [14]. Therefore, the objective of this study was to estimate the potential contribution of rooftop RWH from large public institutions to supplement the potable water demand, and relate the rainwater harvest to city level consumption and consumption at the individual institution.

## 2. Materials and Methods

### 2.1. Study Area

The study was conducted in Addis Ababa, located at 9°1′48″ N and 38°44′24″ E with a total boundary area of 540 km^2^ and a population of more than 3.434 million [8], growing at an annual rate of 3.8% [13]. The climate is warm and temperate with a mean daily temperature of 22 °C. The city’s water supply represents an annual consumption of 152.114 million m^3^, abstracted from ground water (51%) and surface water (49%) sources [14]. When loss in the distribution network is subtracted, the estimated water supplied is 94 L per person per day [14]. However, in recent years, owing to the high rate of urbanization coupled with industrial development and population growth [33], as well as change in precipitation patterns, the available water to satisfy the water demand has radically decreased, representing a 41% deficit in 2016 [14].

### 2.2. Rooftop Rainwater Harvesting

The large public institutions in Addis Ababa were identified from publicly available lists of existing large public institutions, and grouped into 11 categories, as shown in Table 1. It should be noted that, due to their size, the three campuses of Addis Ababa University are presented as individual categories (College of natural science, Addis Ababa institute of Technology (AAiT), and Ethiopian institute of Architecture, Building Construction and City Development (EiABC)). For the first eight categories, 25–30% representative institutions were selected for ArcGIS digitalization, while all buildings on the three campuses of Addis Ababa University (AAU) were digitalized. 

**Figure 1:**
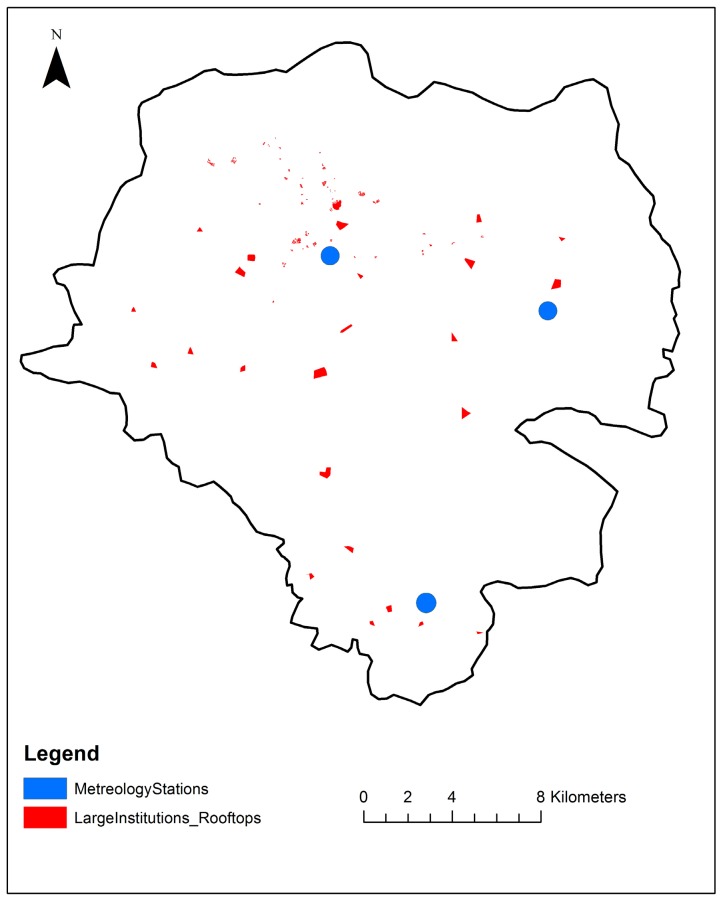
Distribution of the 588 digitalized rooftops used in this study to represent large public institutions in Addis Ababa.

To estimate the average rooftop size, the rooftops of the identified 25–30% representative institutions were first located on an online Google base map from August 2017, which was added to a boundary map of Addis Ababa on ArcGIS (version 10.3.1) Based on the standard methods of the Environmental Systems Research Institute (ESRI), the rooftops of the representative large public institutions were then digitalized and their areas calculated using the Calculate Geometry tool in ArcGIS. After digitalizing was completed, 588 digitized rooftops (Figure 1) were investigated, and the average rooftop areas of the representative institutions were then computed and used to estimate the average rooftop area for each category (Table 1).

### 2.3. Rainfall Data Analysis

**Figure 2:**
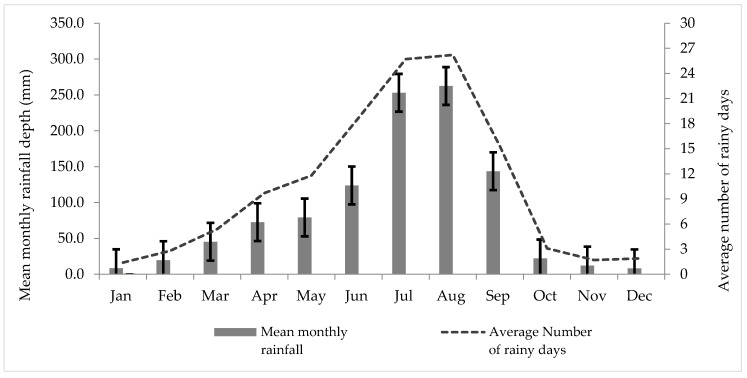
Ten-year (2006–2015) mean monthly rainfall depth (R), standard deviation (the heavy black I-bars), and mean number of rainy days of Addis Ababa from three rain gage stations.

A ten-year monthly rainfall dataset for the period 2006–2015, based on data from three rain gage stations (Bole, Addis Ababa observatory, and Akaki) within Addis Ababa, was obtained from the National Meteorology Agency of Ethiopia (NMA) [12] in digital form and further analyzed in a spreadsheet (Figure 2). The mean annual rainfall of the three stations was used to quantify the potential rooftop RWH from the targeted large public institutions. The average number of rainy days per year was computed from the same rainfall dataset and was found to be 121 days.

Statistical variability in monthly rainfall (intra annual) and accumulated annual rainfall (inter annual) were expressed with coefficients of variation, *CV*, using Equation (1).

(1) $$CV=\frac{Sd}{M_{r}}\times 100$$

where, *Sd* is the standard deviation of rainfall (mm) and *M_r_* is the mean rainfall (mm).

Mean monthly potential rooftop RWH (Vrwh) from each category of large public institutions was calculated by multiplying roof areas (Art) (Table 2 with mean monthly rainfalls (R) (Figure 2) and a runoff coefficient (C) of 0.85 was employed to account for evaporation loss and possible first flush diversion [34] using Equation (2) (modified after Ghisi et al. [23]), resulting in the values shown in Table 3.

(2) Vrwh=R ×Art ×C

### 2.4. Water Supply Data

To assess the potential contribution of rooftop RWH from large public institutions, data for the monthly water supply to the city was collected from the Addis Ababa Water and Sewerage Authority (AAWSA) [11] (Table 2). Similarly, to investigate the potential contribution at the individual public institution level, water consumption data of each of the digitalized institution (Table 1) was collected from their water bill, and summarized in Table 4.

## 3. Results

### 3.1. Rainfall Variability

The 10 years of rainfall statistics (Figure 2) provide the basis for assessing the RWH potential. The variation in rainfall between months is significant, with a CV varying from 90% in the year with the most even distribution to 117% in the year with the largest difference between the wettest August and driest month December, reflecting a climate with strong seasonality in rainfall. If considering only the four wet season months (June through September), the monthly variation with CVs ranges between 36% and 67%. Regarding the variation in total rainfall between years, this is quite modest, with a CV of 13%, reflecting a rather stable rainfall pattern. It should be noted that the lowest annual recordings are the most recent (871 mm in 2015 when compared with 1188 mm in 2006), indicating a possible reduced rainfall, pointing to the need for increasing resilience in the water supply.

### 3.2. Potential of Rooftop RWH from Large Public Institutions to Supplement the Water Supply of Addis Ababa

A summary of the monthly water consumption data of targeted large public institutions is presented in Table 4.

**Figure 3:**
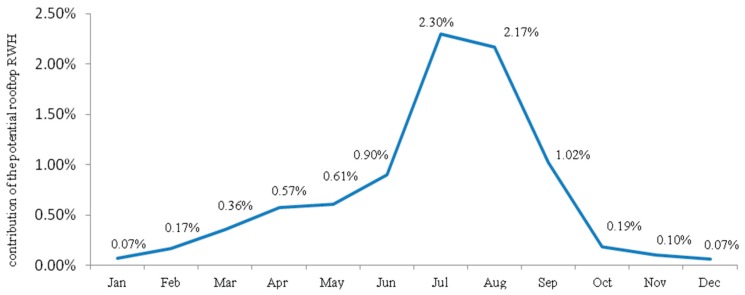
Contribution of rooftop RWH from large public institutions to supplement water supply at the city level.

The estimated potential of rooftop RWH from large public institutions to supplement the water supply at the city level is shown in Figure 3. The accumulated annual contribution amounts to only 0.71% of the actual supply. Even in the rainy season the harvest potential can contribute less than 2.5% of the actual municipal water supply.

For example, in July, 164,817 m^3^ of the potable water can be replaced with water harvested from large public institutions, corresponding to the monthly water demand of some 1637 people. It should be stressed that this is the maximum quantitative volume, not considering restrictions associated with tank size, water losses, water pollution, or other issues likely to reduce the volume that can be attained in practice.

### 3.3. Contribution of Potential Rooftop RWH from Large Public Institutions to Supplement Their Water Supply

Assuming the rainwater harvested is to be consumed by the institution itself, rather than provided to the city, the coverage degree reaches significant levels, as presented in Table 5. In the rainy season, the coverage in several cases surpasses 100%, while the dry season coverage is still, at large, insignificant.

From a pure quantitative point of view, the comparison between RWH and water consumption shows that of the total eleven categories of large public institutions, six of them can replace 100% of their water supply in July and August, and three of them can also do so in September (Table 4). Excess water can be stored for later use if storage facilities are available. Water quality issues will have to be addressed if RWH is to replace all purposes.

## 4. Discussion

The rather low coefficient of variability in rainfall in the wet season from June through September between different years, as also reported by Engida [35], points to reliable conditions for RWH and a guarantee for the return of investment. The steady rainfall conditions in the wet season allow for an efficient replacement of other freshwater sources [36], even with a small storage tank, e.g., relieving the pressure on the groundwater. Regarding the potential of the transfer of excess water from the wet season to later use in the dry season, this relies exclusively on the size of the storage tank, and thus on investment power which could be particularly difficult in low economy institutions, as also reported by Abdulla and Al-Shareef [31]. To be resilient and as a mitigation measure for the existing water supply shortage and climate change problems with a tendency to more frequent droughts [17], the large public institutions in Addis Ababa may install a series of storage tanks, e.g., pre-fabricated polyvinyl tanks, in this way overcoming the limited maximum size of locally available polyvinyl tanks of 25 m^3^ (25,000 L).

As seen from Table 4, the potential for RWH from the considered 588 roof tops of large public institutions to supplement the city of Addis Ababa with freshwater is rather limited. Even in the wettest months, harvested rainwater can replace only 2.3% of 2016’s supply of potable water, corresponding to the water needed by some 1639 persons. On average over the year, RWH can replace 0.71% of today’s water supply. Thus, the potential to narrow the water supply gap at the city level is limited. If all institutions in Addis Ababa were to be involved in rooftop RWH the potential would be approximately four times larger, since the roof tops digitized in this study made up approximately 25% of all large public institutions of the city

Conversely, rooftop RWH from large public institutions can replace a minimum of 0.9% in January to a maximum of 649% in July (Table 5) of the water supply of the large public institutions themselves, showing that the excess rainwater can be stored for later uses. During the rainy months of June through September, about half of the targeted large public institutions could supplement their water supply from rooftop RWH, which saves potable water for the institutions. This implies that if each of the large public institutions is involved in rooftop RWH, it will supplement the potable water from a maximum of 649% to a minimum of 9.9% during the rainy months and from a maximum of 94% to a minimum of 0.9% during the dry months, implying that the potable water supplied to the institutions will be minimized by 2% in the rainy months and from 0.07% (January and December) to 0.61% (May) in the dry months, as nearly 2% of the water supply goes to larger public institutions (comparison of Table 2 and Table 3). Accordingly, an equivalent quantity of potable water could be transferred to localities suffering from a critical water shortage with a potential contribution to narrow the existing water supply deficit of Addis Ababa. In agreement with this, studies conducted in Brazil [16] and Denmark [37] report that RWH potentially contributes up to 69% of the water supply, though the present study reported the potential of RWH from only the rooftops of large public institutions as they are assumed to serve as frontrunners to bring other roof owners into RWH. A study conducted in Nigeria by Aladenola and Adeboye [15] also reports that RWH can considerably contribute to supplementing the water supply, except for the dry months November, December, January, and February.

The potential rooftop RWH (Table 5) showed that the large public institutions such as technical and vocational colleges, local level administrations and EiABC from June to September and Secondary schools, Addis Ababa city bureaus and utility offices, and health centers in July and August have significant rooftop rainwater to store, which can then be used during the dry days, ranging from 128% in June at EiABC to 649% in July at local level administrations, implying that in addition to saving a noteworthy volume of the potable water, the excess rainwater can be stored and utilized for the later dry months provided there will be adequate storage. The present study argues that for large institutions installing larger sized tanks, this may not be a problem as they should focus on the long-term return of RWH.

Generally, rooftop RWH may contribute to minimize the shortage of water supply in Addis Ababa, as the existing water supply sources are vulnerable to the extended dry months and climate change, as already witnessed in Addis Ababa in 2015 [11]. Consistent with this, Kuczera [38] indicates that RWH could minimize the vulnerability of the water supply in urban areas. In addition, RWH will reduce the pressure on groundwater resources as a significant volume of rainwater can be harvested from rooftops, which could result in a potable water save. The increased groundwater will be a reserve for the dry season supply as 51% of the potable water supply of Addis Ababa is from groundwater resources [11]. In agreement with this, a study conducted in Iran shows that groundwater resources decrease due to inappropriate usage and over exploitation [36]. The large public institutions with excess rainwater harvest may take the role of demonstration by establishing water kiosks, as has been done at health centers in rural Rwanda [39]. Interestingly, hospitals make up the largest category in Addis Ababa (Figure 2), and could similarly to the case of Rwanda, serve as centers for water distributors and information centers on healthy water practices, including water quality aspects on the use of water harvested from rooftops. However, if the hospitals use the harvested water themselves, nothing will be left to sell (Table 5). In that case, the technical and vocational colleges/schools hold a larger potential, since they harvest a water surplus (Table 5), which could be sold. Guidance on the construction of RWH could be provided by these colleges/schools. To sustainably utilize rooftop RWH as an alternative source of water supply, the Addis Ababa city administration should encourage RWH by helping the residents with installation costs (e.g., if the residents pay 30%, the city pays 70% of the storage installation costs). The size of storage tank should be investigated in future researches as it may affect the potential contribution of RWH from rooftops. The present study also highlighted that modeling the rainfall-runoff pattern and the potential impact of climate change on Addis Ababa may help to classify the run off coefficient of the local rooftops [40], which could be a limiting parameter for storage tank sizing.

## 5. Conclusions

To investigate the potential contribution of rooftop RWH from large public institutions both at the city and individual institution level, eleven categories of large public institutions were considered in Addis Ababa. From these, the rooftops of 25–30% representative 588 large public institutions were digitalized to obtain the rooftop area. In addition, to quantify the potential volume of rainwater harvesting from each large public institution’s rooftop and to know the rainfall variability, a rainfall dataset from 2006 to 2015 was collected from the Ethiopian national Metreology agency. The water supply of Addis Ababa and the water consumption of each institution were obtained from AAWSA and the water bill of each large public institution, respectively. The findings of the present study showed that the intra rainfall variability ranges between 90 and 117%, while the inter annual variability is 13%, as most of the rain falls within the four rainy months from June through September. Thus, a significant quantity of the potable water can be replaced at the institution level; particularly during the four rainy months, if a strong focus on storing the excess rainwater during such rainy months is given, as the rainfall variability is high during the extended dry months due to the absence of rainfall on many days. At the city level, the large public institutions will have a frontrunner function, especially the hospitals and technical schools. It was estimated that the city can replace up to 2.3% of the potable water supply at the city level through rooftop RWH from large public institutions. Moreover, each of the large public institutions considered in the present study can replace from a maximum of 649% in July to a minimum of 0.9% in December and January of their own potable water supply. Generally, the present study showed that the ability of public institutions to take the role as frontrunners is more promising. 

It is recommended that rooftop RWH in Addis Ababa should be considered as one of the alternative sources of the city’s water supply system considering the enabling water harvesting initiation by the Ethiopian rainwater harvesting association, which could narrow the existing water supply shortage. In addition, the cost-benefit balance, along with the invention of a cheap storage tank, should also be investigated in future researches as they may affect the potential contribution of RWH from rooftops. The present study also highlighted that future studies should emphasize optimal storage tank sizes using alternative construction materials (e.g., polyvinyl, stone/brick masonry) to enable users to clearly know the cost of storage tanks before starting rooftop RWH. It should be noted that the focus of future researches must be in linking stormwater management with RWH as a decentralized stormwater management solution. 

## Figures and Tables

**Table 1:** The categories of large public institutions and the corresponding digitized rooftop areas.

Large Public Institution			Rooftop Area (m^2^)		
No.	Category	Total Existing	Digitalized	Average of Digitalized	Total Estimated
1	Hospitals	13	4	14,273	185,542
2	Secondary schools	27	7	2114	132,651
3	Federal ministries	44	11	2114	93,032
4	Technical and vocational colleges/Schools	10	3	7546	75,457
5	Addis Ababa city bureaus and utility offices	24	6	3059	75,198
6	Local level (Woreda) administration offices	166	10 ^(1)^	453	73,424
7	Sub-city administration offices	10	3	3368	33,683
8	Health centers	23	6	1119	25,729
9	College of natural science	1	1	25,790	25,790
10	AAiT	1	1	23,104	23,104
11	EiABC	1	1	23,103	23,103
	Sum	320	50		766,713

^(1)^ 166 of the Woredas have same rooftop model/size, consequently for convenience only 10 were digitized.

**Table 2:** The 2016 water supply data of Addis Ababa city.

	Jan.	Feb.	Mar.	Apr.	May	Jun.	Jul.	Aug.	Sep.	Oct.	Nov.	Dec.
Monthly water supply (in 1000 m^3^)	123,67	12,588	13,470	13,484	13,924	14,615	11,746	12,942	14,995	12,630	12,711	13,004

**Table 3:** Potential volume of monthly rooftop RWH (m^3^) from each large public institution.

Category of Institution ^(2)^	Jan.	Feb.	Mar.	Apr.	May	Jun.	Jul.	Aug.	Sep.	Oct.	Nov.	Dec.
1	1309	3091	7129	11,434	12,475	19,509	39,885	41,383	22,647	3470	1908	1293
2	936	2210	5096	8175	8919	13,948	28,515	29,586	16,191	2481	1364	925
3	656	1550	3574	5733	6255	9782	19,999	20,750	11,355	1740	957	648
4	532	1257	2899	4650	5073	7934	16,221	16,830	9210	1411	776	526
5	518	1223	2821	4525	4937	7720	15,784	16,376	8962	1373	755	512
6	531	1253	2889	4634	5056	7907	16,165	16,772	9179	1406	773	524
7	238	561	1294	2076	2265	3542	7241	7513	4111	630	346	235
8	182	429	989	1586	1730	2705	5531	5739	3141	481	265	179
9	182	430	991	1589	1734	2712	5544	5752	3148	482	265	180
10	163	385	888	1424	1553	2429	4967	5153	2820	432	238	161
11	163	385	888	1424	1553	2429	4966	5153	2820	432	238	161

^(2)^ Refer to Table 1 for category names.

**Table 4:** Summary of the monthly water consumption data of targeted large public institutions.

Category of Institution ^(3)^	Monthly Water Consumption Data (in 1000 m^3^)											
	Jan.	Feb.	Mar.	Apr.	May	Jun.	Jul.	Aug.	Sept.	Oct.	Nov.	Dec.
1	75.0	70.3	67.8	57.6	56.5	58.7	108.2	89.1	84.6	84.7	100	92.9
2	21.9	21.1	19.2	21.6	25.8	35.3	21.2	19.3	19.3	14.9	20.3	21.4
3	77.7	82.4	86.1	88.7	60.6	99.2	68.2	53.5	53.7	50.7	58.3	71.8
4	5.1	4.8	5.3	5.6	5.4	5.2	5.04	4.9	4.8	5	5.2	5.7
5	27	16.5	15.6	16.2	10.5	12.2	5.9	19.9	20.1	12.6	16.3	11.6
6	3.7	3.3	3.2	2.8	3.2	3.3	2.5	2.7	2.5	2.3	2.5	2.5
7	14.7	16.2	12.4	15.6	13.6	10.4	18.5	13.2	9.8	7.5	8.9	10.0
8	5.3	5.3	4.6	4.9	5.3	5.6	5.2	5.0	5.4	5.5	5.9	5.7
9	15.5	8.6	11.5	14.8	14.8	14.8	14.9	16.0	12.2	9.4	11.9	14.3
10	10.7	10.7	9.7	9.9	8.9	8.1	11.2	8.2	7.9	8.5	7.6	11.8
11	4.9	5.0	4.1	3.2	4.3	1.9	8.8	1.1	1.02	4.1	4.3	5.0

^(3)^ Refer to Table 1 for category names.

**Table 5:** Water supplement of large public institutions in Addis Ababa due to potential rooftop RWH from their rooftops.

Large Public Institution	Water Supply Supplement from Rooftop RWH (%)											
	Jan.	Feb.	Mar.	Apr.	May	Jun.	Jul.	Aug.	Sep	Oct	Nov.	Dec.
Hospitals	1.8	4.4	10.5	19.9	22	33	37	46	27	4.1	1.9	1.4
Secondary schools	4.3	10.5	26.6	38	35	39	134	154	84	16.6	6.7	4.3
Federal ministries	0.9	1.9	4.2	6.5	10	9.9	29	39	21	3.4	1.6	0.9
Technical and vocational colleges/Schools	10.6	26	55	83	94	152	322	342	192	28	15	9.2
Addis Ababa city bureaus and utility offices	1.9	7.4	18.0	28.0	47	63	266	82	45	10.9	4.6	4.4
Local level administrations (Woredas)	14.5	37.7	92	164	160	238	649	632	369	61	31	21
Sub-city administration offices	1.6	3.5	10.4	13.3	17	34	39	57	42	8.4	3.9	2.3
Health centers	3.5	7.8	21.6	32.6	33	48	106	115	58	8.7	4.5	3.1
College of natural science	1.2	5.0	8.7	11	12	18	37	36	26	5.1	2.2	1.3
AAiT	1.5	3.6	9.1	14	17	30	44	63	36	5.1	3.1	1.4
EiABC	3.3	7.7	21.6	45	36	128	568	500	277	10.4	5.5	3.2

## References

[1] Gleick P.H. (1996). Basic water requirements for human activities: Meeting basic needs. Water Int..

[2] Wheida E., Verhoeven R. (2007). An alternative solution of the water shortage problem in Libya. Water Resour. Manag..

[3] Fang C.L., Bao C., Huang J.C. (2007). Management implications to water resources constraint force on socio-economic system in rapid urbanization: A case study of the Hexi Corridor, NW China. Water Resour. Manag..

[4] Seckler D., Barker R., Amarasinghe U. (1998). Water scarcity in the twenty-first century. Int. J. Water. Resour. Dev..

[5] United Nations (2015). The Millennium Development Goals Report 2006.

[6] Haque M.M., Rahman A., Samali B. (2016). Evaluation of climate change impacts on rainwater harvesting. J. Clean. Prod..

[7] Central Statistical Agency of Ethiopia (1984). Population and Housing Census of Ethiopia.

[8] Federal Democratic Republic of Ethiopia Central Statistical Agency (2013). Population Projection of Ethiopia for All Regions from 2014–2017.

[9] Viste E., Korecha D., Sortenberg A. (2013). Recent drought and precipitation tendencies in Ethiopia. Theor. Appl. Clim..

[10] Samela C. (2016). DEM-Based approaches for the delineation of flood-prone areas in an ungauged basin in Africa. J. Hydrol. Eng..

[11] Addis Ababa Water and Sewerage Authority (2016). Surface and Groundwater Supply Data.

[12] National Metrology Agency (2016). Monthly Rainfall Data of Addis Abeba.

[13] World Bank (2015). Enhancing Urban Resilience in Addis Ababa: Resilient Cities Program.

[14] Addis Ababa Water and Sewerage Authority (2011). Business Plan from 2011 to 2020: Final Report.

[15] Steffen J., Jensen M., Pomeroy C.A., Burian S.J. (2013). Water supply and stormwater management benefits of residential rainwater harvesting in US cities. J. Am. Water Resour. Assoc..

[16] Ward S.L. (2010). Rainwater Harvesting in the UK: A Strategic Framework to Enable Transition from Novel to Mainstream. Ph.D. Thesis.

[17] Zhang Y., Chen D., Chen L., Ashbolt S. (2009). Potential for rainwater use in high-rise buildings in Australian cities. J. Environ. Manag..

[18] Herrmann T., Schmida U. (2000). Rainwater utilization in Germany: Efficiency, dimensioning, hydraulic and environmental aspects. Urban Water.

[19] Alem G. (1999). Rainwater Harvesting in Ethiopia: An Overview.

[20] Kahinda J.M., Taigbenu A.E. (2011). Rainwater harvesting in South Africa: Challenges and opportunities. Phys. Chem. Earth.

[21] Ibrahim M.B. (2009). Rainwater harvesting for urban areas: A success story from Gadarif city in central Sudan. Water Resour. Manag..

[22] Aladenola O., Adeboye O.B. (2010). Assessing the potential for rainwater harvesting. Water Resour. Manag..

[23] Ghisi E., Montibeller A., Schmidt R.W. (2006). Potential for potable water savings by using rainwater: An analysis over 62 cities in southern Brazil. Build. Environ..

[24] Amin M.T., Alazba A.A., Elnesr M.N. (2013). Adaptation of climate variability/extreme in arid environment of the Arabian peninsula by rainwater harvesting and management. Int. J. Environ. Sci. Technol..

[25] Mehrabadi M.H.R., Saghafian B., Fashi F.H. (2013). Assessment of residential rainwater harvesting efficiency for meeting non-potable water demands in three climate conditions. Resour. Conserv. Recycl..

[26] Pandey D.N., Gupta A.K., Anderson D.M. (2003). Rainwater Harvesting as an Adaptation to Climate Change. Curr. Sci..

[27] Sazakli E., Alexopoulos A., Leotsinidis M. (2007). Rainwater harvesting, quality assessment and utilization in Kefalonia Island, Greece. Water Res..

[28] Mendez C.B., Klenzendorf J.B., Afshar B.R., Simmons M.T., Barrett M.E., Kinney K.A., Kirisits M.J. (2011). The effect of roofing material on the quality of harvested rainwater. Water Res..

[29] Sample D.J., Liu J. (2014). Optimizing rainwater harvesting systems for the dual purposes of water supply and runoff capture. J. Clean. Prod..

[30] Wang W.C., Xu D.M., Chau K.W., Lei G.J. (2014). Assessment of river water quality based on theory of variable fuzzy sets and fuzzy binary comparison method. Water Resour. Manag..

[31] Abdulla F.A., Al-Shareef A.W. (2006). Assessment of rainwater roof harvesting systems for household water supply in Jordan. Integrated Urban Water Resources Management.

[32] Zaizen M., Urakawa T., Matsumoto Y., Takai H. (1999). The collection of rainwater from dome stadiums in Japan. Urban Water.

[33] Kasa L., Zeleke G., Alemu D., Hagos F., Heinimann A. (2011). Impact of Urbanization of Addis Abeba City on Peri-Urban Environment and Livelihoods.

[34] Thomas T.H., Martinson D.B. (2007). Roof Water Harvesting: A Handbook for Practitioners.

[35] Engida M. (1999). Annual rainfall and potential evapo-transpiration in Ethiopia. Ethiop. J. Natl. Resour..

[36] Gholami V., Chau K.W., Fadaee F., Torkaman J., Ghaffari A. (2015). Modeling of groundwater level fluctuations using dendrochronology in alluvial aquifers. J. Hydrol..

[37] Mikkelsen P.S., Adeler O.F., Albrechtsen H.J., Henze M. (1999). Collected rainfall as a water source in Danish households: What is the potential and what are the costs?. Water Sci. Technol..

[38] Kuczera G. (2007). Regional Impacts of Roof Water Harvesting—Supplementing Public Water Supply.

[39] Huttinger A., Brunson L., Moe C.L., Roha K., Ngirimpuhwe P., Mfura L., Kayigamba F., Ciza P., Dreibelbis R. (2017). Small water enterprise in Rural Rwanda: Business development and year-one performance evaluation of nine water kiosks at health care facilities. Int. J. Environ. Res. Public Health.

[40] Chau K.W. (2017). Use of meta-heuristic techniques in rainfall-runoff modeling. Water.

